# Prevalence and WGS-based characteristics of MRSA isolates in hospitals in Shanghai, China

**DOI:** 10.3389/fmicb.2022.1002691

**Published:** 2022-11-03

**Authors:** Hongzhi Zhang, Liang Tian, Taiyao Chen, Wenjie Chen, Yilin Ge, Jing Bi, Zhixin Fang, Min Chen

**Affiliations:** Department for Pathogen Identification, Shanghai Municipal Center for Disease Control and Prevention, Shanghai, China

**Keywords:** methicillin-resistant *S. aureus* (MRSA), whole genome sequencing, MLST, cgMLST, antimicrobial resistance

## Abstract

Methicillin-resistant *Staphylococcus aureus* (MRSA) isolates remain a serious threat to global health despite a decrease in MRSA infections since 2005. MRSA isolates exhibit great diversity worldwide, and their lineages show geographic variation. In this study, we used whole genome sequencing (WGS) to analyze antibiotic resistance genes and virulence genes, *spa*, staphylococcal cassette chromosome *mec*, sequence types (STs), and core genome multilocus sequence typing (cgMLST) of MRSA isolates from patients and environmental surface in hospitals in China to determine their prevalence and molecular traits. The highest number of infections by MRSA isolates was observed in patients aged ≥60 years (69.8%, *P* < 0.05). We identified a total of 19 STs from 162 MRSA isolates from patients. A significant increase was observed in the incidence of ST764-t002-II MRSA infection, which is replacing ST5-t002-II MRSA as the predominant ST. Similarly, isolates from environmental surface were predominantly ST764-t002-II (47%). Notably, most ST764 isolates (97.7%) carried *seb*, but not arginine catabolic mobile element (ACME), which differed from ST764 isolates in Japan and Thailand. The potential danger of spreading requires rigorous surveillance of emerging ST764 MRSA isolates. We also found higher resistance to seven antimicrobials [OXA, cefoxitin (FOX), ERY, CFZ, ciprofloxacin (CIP), levofloxacin (LEV), and moxifloxacin (MXF)]. Resistance to gentamicin (38.3%), tetracycline (55.9%), and minocycline (41.5%) were also common. Phenotypic resistance to antimicrobials was associated with resistance genes to its content, and cgMLST clustering suggested a strong link between these strains. Overall, our findings revealed the prevalence and molecular characteristics of MRSA isolates in Shanghai, China, providing a theoretical reference for preventing and controlling clonal transmission of MRSA isolates in hospitals in China.

## Introduction

*Staphylococcus aureus* is an important Gram-positive bacterium that is commonly associated with significant morbidity, hospital mortality, length of stay, and economic burden (Centers for Disease Control and Prevention, 2019). In 1959, methicillin was introduced to treat infections caused by penicillin-resistant *S. aureus*. However, by 1961, *S. aureus* isolates had acquired resistance to methicillin (methicillin-resistant *S. aureus*, MRSA) (Jevons, 1961), and MRSA isolates were soon transmitted worldwide. Today, MRSA is a serious global health threat and responsible for a range of infections, from skin and wound infections to pneumonia and septicemia.

Since 2005, a parallel decrease in MRSA infections has been confirmed in the United States and multiple European countries (Dantes et al., 2011; de Kraker et al., 2013). Notably, MRSA prevalence decreased from 69.0% in 2015 to 31.4% in 2019 in China (Hu et al., 2020). However, this is still higher than other common multidrug-resistant bacteria, and MRSA is considered a major cause of clinical infections and a severe threat to both communities and healthcare facilities (Tong et al., 2015).

Methicillin-resistant *Staphylococcus aureus* isolates exhibit great global diversity (Turner et al., 2019), and their lineages appear to vary geographically worldwide (Chambers and DeLeo, 2009). In China, ST239-SCCmec III/II and ST5-SCCmec II are the prevalent hospital-acquired (HA)-MRSA clones (Chen and Huang, 2014), while ST59-SCCmec IV/V is the prevalent clone associated with community-associated (CA)-MRSA (Chuang and Huang, 2013). ST72 was reported as dominant in Korea, ST8 or ST30 in Japan, and ST59 in Taiwan (). ST22 and ST36 were reported as predominant strains in the United Kingdom in the late 1990s (Murchan et al., 2004; Knight et al., 2012) and have also been reported as the predominant strains in continental Europe (Johnson, 2011). Strain ST22 has been reported as gradually overtaking ST239, another widely-distributed strain found in Europe, the Middle East, Asia, and the Pacific (Rolo et al., 2012; Xie et al., 2016). MRSA USA300 has been identified in the United States, where its prevalence varies widely according to geographic location (David et al., 2014).

Of note, MRSA has the capacity for gaining new antimicrobial resistance traits by acquiring mobile genetic elements carrying resistance genes (Gould et al., 2012; Amoako et al., 2016), such as those conferring resistance to trimethoprim (*drfA* and *drfK*), erythromycin (ERY; *ermC*), clindamycin (CLI; constitutively expressed *merC*), and tetracyclines (TETs; *tetK* and *tekL*) (Malachowa and DeLeo, 2010). Furthermore, likely reflecting the stress of antibiotics, MRSA also acquires resistance to other antibiotics.

Methicillin-resistant *Staphylococcus aureus* colonization increases the risk of infection. MRSA strains can colonize almost any surface, such as floors, white coats, keyboards, etc., and colonization can persist for a long time. Furthermore, colonization is not static. MRSA strains evolve by acquiring genes conferring virulence or resistance (Azarian et al., 2016). For example, ST764 MRSA is a hybrid variant of ST5 HA-MRSA with the characteristics of CA-MRSA from the acquisition of two mobile genetic elements, arginine catabolic mobile element (ACME) II and SaPInn54, which enhance the expression of cytolytic peptide genes (Takano et al., 2013).

Over the past decades, many molecular typing methods, including pulsed-field gel electrophoresis (PFGE), multilocus sequence typing (MLST), *spa* typing, and SCCmec typing, have been used to determine MRSA epidemiology. Although PFGE is considered the “gold standard” (Li et al., 2019), it is a low-throughout technique, and it is difficult to share or compare the results between different laboratories. Furthermore, most ST398 isolates cannot be typed with PFGE using *Sma*I due to methylation of the *Sma*I site (Bens et al., 2006). Recently, whole genome sequencing (WGS) has become a more accessible and affordable tool for bacterial typing. Furthermore, WGS data can be used to analyze ST, *spa*, SCCmec, core genome (cg) MLST, etc. All of these typing methods are employed in phylogenetic analysis and epidemiological investigations.

Although many studies on the molecular characteristics of MRSA have been reported worldwide (Turner et al., 2019), and there have been many epidemiological studies of MRSA in China, they mainly focused on a subset of MRSA from specific infection type source, such as blood, HA-MRSA, or CA-MRSA (). The well-known understand of entire MRSA in hospital in China is still lack. Furthermore, continuous monitoring of changes in MRSA epidemiology is crucial because the predominant strains are continuously evolving, and the predominant sequence types (STs) keep changing, leading to challenges in hospital infection control. Therefore, we performed a systematic surveillance study using WGS on MRSA isolates from Chinese patients and environmental surface to analyze antibiotic resistance genes and antibiotic sensitivity, virulence genes, *spa*, SCCmec, STs, and core genome multilocus sequence typing (cgMLST). Our findings provide epidemiological evidence for the prevention and control of *S. aureus* infections and transmission in hospitals.

## Materials and methods

### Bacterial isolates

This study analyzed 162 *S. aureus* isolates obtained from five general hospitals in Shanghai during 2017–2019. All samples were collected, then, *S. aureus* isolates were detected from these samples in hospitals. At last identified *S. aureus* isolates were sent to our laboratory for further research.

All isolates were confirmed as MRSA carrying *mecA* and showing resistance to oxacillin (OXA). Furthermore, we also analyzed 51 *S. aureus* isolates from samples taken from the environmental surface in these hospitals, including substance that came into contact with patients, medical staff, and devices. CA-MRSA was defined as an isolate from either an outpatient or an inpatient within 48 h of hospitalization. The first *S. aureus* isolate obtained from each patient was analyzed.

### Antimicrobial susceptibility testing

All MRSA isolates underwent antimicrobial susceptibility testing using the broth dilution method and Gram-positive panels (Zhuhai Meihua Medical Technology Co., Ltd., Guangzhou, China) according to the Clinical and Laboratory Standards Institute (2019) guidelines [23]. The 13 drugs included oxacillin (OXA; 0.25–16 μg/mL), cefazolin (CFZ; 0.5–32 μg/mL), gentamicin (GEN; 1–64 μg/mL), ERY (0.25–16 μg/mL), TET (0.5–32 μg/mL), minocycline (MIN; 0.06–64 μg/mL), trimethoprim-sulfamethoxazole (T/SUL) (0.12/2.38–8/152 μg/mL), rifampin (RIP; 0.12–128 μg/mL), lincomycin (LNE; 0.25–32 μg/mL), doxycycline (DOX; 0.06–64 μg/mL), moxifloxacin (MXF; 0.06–64 μg/mL), and teicoplanin (TEC; 0.12–128 μg/mL). *S. aureus* American Type Culture Collection (ATCC) 29213 was used as the control for the antimicrobial susceptibility testing.

### Genomic DNA extraction and whole genome sequencing

Overnight cultures of *S. aureus* isolates were harvested, and DNA was extracted using a DNeasy Blood and Tissue Kit (Qiagen, Germantown, MD, USA) according to the manufacturer’s protocol, except that the bacterial cells were paralyzed with lysostaphin for 30 min at 37°C and the proteinase K treatment was extended to 30 min. The resultant DNA concentration, quality, and integrity were assessed using a Qubit Fluorometer (Invitrogen, USA) and a Nanodrop Spectrophotometer (Thermo Scientific, USA). Sequencing libraries were generated using a TruSeq DNA Sample Preparation Kit (Illumina, USA). Then, next-generation sequencing was performed using an Illumina HiSeq platform (Illumina). Finally, the reads were trimmed and assembled using CLC Genomics Workbench v7.0 software (CLC Bio, Aarhus, Denmark). The assembled contigs were exported as raw sequencing reads that was quality checked using FastQC v0.11.2 and trimmed using Trimmomatic v0.36. Subsequently, the trimmed reads were *de novo* assembled into contigs using BioNumerics v7.6 (Applied Maths, Kortrijk, Belgium), and the assembled sequence was used for further analysis.

A total of 199 MRSA isolates, including 162 MRSA isolates from patients and 35 MRSA isolates from environmental surface, two ST764-MRSA-II isolates (SRR16971216 and SRR16971212) from Thailand isolates from National Centre for Biotechnological Information (NCBI) were analyzed using WGS.

### Multilocus sequence typing, SCCmec, *spa*, toxin gene profiles, and resistance gene profiles

Sequence types were assigned using BioNumerics software according to the classical seven housekeeping loci MLST scheme (*arc*, *aroE*, *glpF*, *gmk*, *pta*, *tpi*, and *yqiL*) (Enright et al., 2000), and sequence data of the isolates were extracted from their genomic data.

Virulence-associated genes extracted from WGS data using BioNumerics software were added to the Virulence Factor Database [Ministry of Health (MOH) Key Laboratory of Systems Biology of Pathogens (Institute of Pathogen Biology, Beijing, China)].^1^ The resistance gene profiles were analyzed using the WGS data by online flat^2^, which integrated data from CARD^3^ and ResFinder.^4^

### Core genome multilocus sequence typing characterization

Core genome multilocus sequence typing typing was conducted based on the WGS tool of BioNumerics v7.6 (Applied Maths) with integrated 1,861 loci cgMLST scheme. The threshold values for interpreting clonality with cgMLST were as follows: ≤8 allelic differences, related; 9–29 allelic differences, possibly related; and ≥30 allelic differences, unrelated (Cunningham et al., 2017).

### Detection of the arginine catabolic mobile element

An alignment of the USA300 acquired-ACME related genes was carried, including *acrA*, *acrB*, *acrC*, *acrD*, *opp3*, and *ccrC* (GenBank accession no.: CP000255.1).

### Statistical analysis

All statistical analyses were performed using chi-square tests, where *P* < 0.05 was considered statistically significant.

## Results

### Clinical characteristics of methicillin-resistant *Staphylococcus aureus* isolates from patients infections in Shanghai, China

We analyzed a total of 162 confirmed MRSA isolates from patient infections in five general hospitals (HA, HB, HC, HD, and HE) during 2017–2019 in Shanghai, China (Table 1). The hospitals included two upper first-class hospitals (HE and HB), two upper second-class hospitals (HA and HD), and one middle second-class hospital (HC). These hospitals have been differentiated according to scale of hospitals, capacity of scientific research, etc. The upper first-class hospitals were the maximum scale and the strongest scientific research level, followed by upper second-class hospitals and middle second-class hospitals. Each case was represented by a single isolate. The highest number of cases was observed in patients aged ≥60 years (69.8%, *P* < 0.05). Furthermore, the number of patients aged ≥60 years was the highest for all five hospitals. Relative to cases from HB, infections in the other four hospitals were more likely to be pulmonary (*P* < 0.05). Although the number of infections in HA, HB, and HC was higher than that in HE, the proportions determined as CA-MRSA were higher in HE. Furthermore, there was no difference between the number of HA-MRSA and CA-MRSA strains in this study. A total of 51 *S. aureus* isolates were from hospital environmental surface, of which 35 were confirmed as MRSA isolates due to the presence of *mecA* and OXA resistance. The highest number of MRSA isolates was obtained from items in contact with patients (45.1%, *P* < 0.05). The distribution of MRSA isolates differed among the hospitals. In HE, the number of MRSA isolates from items in contact with patients was the highest (89.5%, *P* < 0.05); however, the number of MRSA isolates from items in contact with patients and from medical devices in HA was the same (both 44.4%). Overall, the number of MRSA isolates from medical devices was the lowest (7.8%, *P* < 0.05).

**TABLE 1:** Clinical characteristics of methicillin-resistant *Staphylococcus aureus* (MRSA) infections during 2017–2019 in Shanghai, China.

Characteristics		Hospitals				
**Age group, year**	Total (n)/%	HA (n)/%	HB (n)/%	HC (n)/%	HD (n)/%	HE (n)/%
0–4	10 (6.2%)	2 (7.2%)	1 (4.5%)	5 (10.9%)	1 (3.0%)	1 (3.0%)
5–19	4 (2.5%)	2 (7.2%)	0	0	1 (3.0%)	1 (3.0%)
20–39	9 (5.5%)	1 (3.6%)	3 (13.4%)	4 (8.7%)	1 (3.0%)	0
40–59	26 (16.1%)	3 (11.5%)	7 (31.8%)	8 (17.4%)	1 (3.0%)	7 (21.2%)
60+	113 (69.8%)^a^	20 (71.4%)^a^	11 (50.0%)^a^	29 (63.0%)^a^	29 (87.8%)^a^	24 (72.7%)^a^
Sex-male	101 (62.3%)	13 (46.4%)	15 (68.2%)	30 (65.2%)	18 (54.5%)	20 (60.6%)
**Primary infection source**						
Skin or soft tissue	24 (14.5%)	3 (10.7%)	8 (34.8%)c	8 (17.0%)	3 (8.6%)	2 (6.1%)
Pulmonary	99 (59.6%)^b^	20 (71.5%)^b^	5 (2.2%)	27 (57.5%)^b^	21 (60%)^b^	26 (78.85)^b^
Surgical site	10 (6.0%)	0	1 (4.3%)	2 (4.3%)	6 (17.1%)	1 (3.0%)
Urinary	6 (3.6%)	3 (10.7%)	1 (4.4%)	0	1 (2.9%)	1 (3.0%)
Blood	6 (3.6%)	0	3 (13.0%)	3 (6.4%)	0	0
Other sites	17 (10.2%)	2 (7.1%)	5 (21.7%)	7 (14.95)	0	3 (9.1%)
**Location of acquisition**						
Hospital	87 (53.7%)	20 (71.4%)^d^	15 (68.1%)^d^	29 (63.0%)^d^	7 (21.2%)	16 (48.5%)
Community	75 (46.3%)	8 (28.6%)	7 (31.8%)	17 (37.0%)	26 (78.8%)^e^	17 (51.5%)
**Environments source**	MRSA (n)/%					
Items contacted with patients	23 (45.1%)^f^	4 (44.4%)	1 (7.15%)	1 (20%)	0/4	17 (89.5%)^g^
Items contacted with medical staffs	8 (15.7%)	0	4 (28.6%)	0/5	1 (25%)	3 (15.8%)
Medical device	4 (7.8%)	4 (44.4%)	0/14	0/5	0	0

Lowercase letters (a–g) indicate a significant difference (*P* < 0.05) within the same characteristics among different hospitals.

### Genetic characteristics of methicillin-resistant *Staphylococcus aureus* isolates across study periods

**FIGURE 1:**
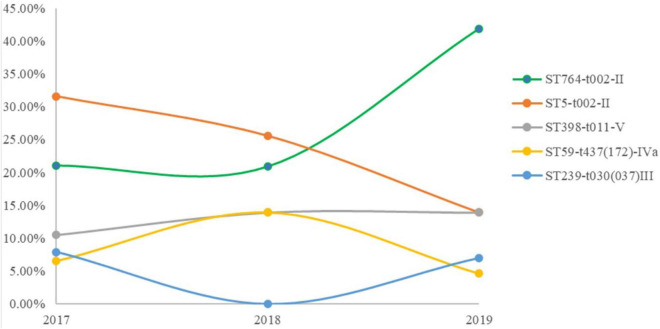
Minimal spanning tree based on multilocus sequence typing (MLST) of methicillin-resistant *Staphylococcus aureus* (MRSA) isolates. All 14 STs are represented by a circle with color. Non-typeable isolates were excluded.

We identified 15 different MLST CCs (19 STs) from 162 confirmed MRSA isolates. The vast majority of MRSA isolates belonged to CC5 (54.2%), followed by ST398 (12%), ST59 (9.2%). CC5 included three STs, namely, ST764 (27.2%), ST5 (25.9%), and ST965 (1.2%) (Table 2). Notably, the predominant STs changed during the 3 years (Figure 1). ST5-t002-II was predominant in 2017, ST764-t002-II in 2018, and ST764-t002-II again in 2019, and the number of ST764-t002-II MRSA isolates was obviously higher than that of ST5-t002-II in 2019. There was no difference in the distribution of ST398-t011-V during 2017–2019. Among the MRSA isolates from environmental surface, ST764 was dominant (47%), followed by ST59 (17.65%) and ST5 (11.75%).

**TABLE 2:** Genetic characteristics of methicillin-resistant *Staphylococcus aureus* (MRSA) isolates during 2017–2019 in Shanghai, China.

Characteristics	Total	2017	2018	2019
**CC5 from patients**				
ST764	44 (27.2%)	16 (9.9%)	9 (5.6%)	19 (11.7%)
ST5	42 (25.9%)	25 (15.4%)	11 (6.8%)	6 (3.7%)
ST965	2 (1.2%)	1 (0.6%)	1 (0.6%)	0
SCCmec Type/ST764				
II	35 (21.6%)^a^	9/(5.6%)	9/(5.6%)	17 (10.5%)
V	1 (0.6%)	1 (0.6%)	0	0
Other types	1 (0.6%)	0	0	1 (0.6%)
PVL gene	0			
*tst*	0			
*se* genes	seb/43 (97.7%), sea/0, sec/0, sed/0, sel/0, seq/0, and selk/40 (90.9%)			
SCCmec Type/ST5				
II	30 (18.5%)	24 (14.8%)	10 (6.2%)	6 (3.7%)
PVL gene	0			
*tst*	42 (25.9%)			
*se* genes	seb/0, sea/32 (76.1%), sec/39 (92.8%), sed/0, sel/39 (92.8%), seq/0, and selk/34 (81.0%)			
**ST398 from patients**				
SCCmecV	18 (11.1%)^b^	9 (5.6%)	5 (3.1%)	4 (2.5%)
Other SCCmec types	3 (1.9%)	0	1 (0.6%)	2 (1.2%)
PVL gene	0	0	0	0
*tst*	0	0	0	0
**ST59 from patients**				
SCCmecIVa	13 (8.0%)	5 (3.1%)	6 (3.7%)	2 (1.2%)
PVL gene	2 (1.2%)	0	2 (1.2%)	0
**ST338 from patients**				
SCCmecIVa	2 (1.2%)	1 (0.6%)	0	1 (0.6%)
PVL gene	0	0	0	0
*tst*	0	0	0	0
**PVL gene**	7 (4.3%)	ST88 (1.2%), ST338 (1.2%), ST59 (1.2%), and ST22 (0.6%)		
**MRSA from environments**				
ST764	16 (45.7%)^c^			
ST59	6 (17.1%)			
ST5	4 (11.4%), tst (11.4%)			

Lowercase letters (a,b) indicates a significant difference (*P* < 0.05) between different SCCmec types, and c indicates a significant difference (*P* < 0.05) between different STs.

**FIGURE 2:**
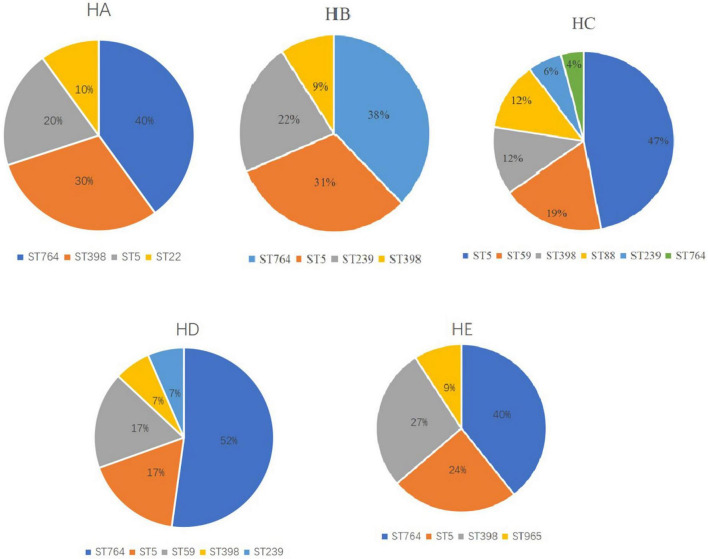
Major sequence types (STs) in each of the five hospitals.

Of note, the major STs were different among the hospitals (Figure 2). ST764 was predominant in four hospitals, followed by ST5. However, ST764 was not major, only 4%, other four STs were ST5 (47%), ST59 (19%), ST398 (12%), and ST88 (12%). This hospital was in a suburb in Shanghai, and the other four hospitals were in urban Shanghai.

The distribution of toxin genes differed among the STs. The predominant STs, including ST764, ST5, and ST398, did not carry Panton–Valentine leucocidin (PVL) genes, but two ST88, two ST338, two ST59, and one ST22 MRSA isolate did. Almost all ST764 MRSA isolates (97.7%) carried *seb*. In contrast, none of the ST5 MRSA isolates carried *seb*. All the ST5 isolates carried *tst*, but ST764 did not. The ST5 isolates also carried *sea* (76.1%), *sec* (92.8%), *sel* (92.8%), and *selk* (81.0%). However, ST764 isolates also carried *selk* (90.0%) (Table 2).

### Antimicrobial resistance gene carriage and phenotypic characteristics

The resistance rate of the 162 MRSA isolates from patients was >60%, showing resistance to OXA, cefoxitin (FOX), ERY, CFZ, ciprofloxacin (CIP), levofloxacin (LEV), and MXF. Most MRSA isolates from patients were susceptible to daptomycin (DAP), RIP, T/SUL, LNE, and TEC, with a resistance rate of <2%. All MRSA isolates from patients were susceptible to vancomycin (VAN).

The resistance rate of the 35 MRSA isolates from the environmental surface was >50%, showing resistance to FOX, OXA, ERY, and CLI. The resistance rate to GEN, CFZ, and TET was 30–50% and <5% to DAP, T/SUL, LNE, and RIP. Furthermore, all 35 MRSA isolates from environmental surface were susceptible to fusidic acid (FD), TEC, and VAN.

All 196 MRSA isolates were phenotypically resistant to OXA and carried *mecA* (100%), while six MRSA isolates was phenotypically susceptible to FOX (96.9% resistance) (Table 3). The β-lactamase-encoding *blaZ* was present in 67.3% (132/196) of the MRSA isolates. The aminoglycoside resistance genes included ant(9)-Ia + aac(6′)-aph(2″) (*n* = 59), ant9-Ia + aac(6′)-aph(2″) + aadD (*n* = 30), ant(6)-Ia + aph(3′)-III (*n* = 20), ant(9)-Ia (*n* = 14), aadD + ant(9)-Ia (*n* = 9), ant(9)-Ia + aac(3)-XI-aac(6′)-aph(2″) + aadD (*n* = 1), ant(6)-Ia + aadD + aac(6)′-aph(2″) (*n* = 1), aac(6′)-aph(2″) (*n* = 2), aph(3″)-III (*n* = 2), aph(2″)-I (*n* = 1), aadD (*n* = 3), ant(9)-Ia + aac(6′)-aph(2′) (*n* = 3), and aadD + ant(9)-Ia/9aph(3′)-III + ant(6)-Ia + aac(6′)-aph(2″) (*n* = 1). Of them, 38.3% showed GEN resistance with minimum inhibitory concentration (MIC) testing. Macrolide resistance genes were observed in 72.4% (142/196) of MRSA isolates, including *erm*(A) (*n* = 75), *lnu*(A) + *erm*(A) (*n* = 26), *erm*(C) (*n* = 16), *erm*(B) (*n* = 16), *lnu*(A) (*n* = 3), *erm*(T) (*n* = 1), *Isa*(E) + *lnu*(B) + *erm*(C) (*n* = 1), *lnu*(A) + *erm*(A) + *erm*(X) (*n* = 1), *erm*(C) + *lun*(A) (*n* = 2), and *msr*(A) + *mph*(C) (*n* = 1). However, 80.6% of the 196 MRSA isolates that were ERY-resistant and identified *via* MIC testing carried resistance genes. The TET resistance genes *tetM* (*n* = 76), *tetK* (*n* = 19), and *tetL* (*n* = 1) were carried in this study, and dual carriage (*tetK* + *tetM*) was observed in two cases. However, 105 MRSA isolates were resistant to TET, as identified using MIC testing. T/SUL resistance genes were uncommon, with only nine *drfG* genes observed, and six MRSA isolates were resistant to T/SUL *via* MIC testing.

**TABLE 3:** Antibiotic resistant genes and resistant phenotype of methicillin-resistant *Staphylococcus aureus* (MRSA) isolates in this study.

Antibiotics	Antibiotic resistant genes/No.	Resistant phenotype/Resistant rate
Beta-lactam	*mecA*/196, *blaZ*/132 ant(9)-Ia + aac(6′)-aph(2″)/59, ant9-Ia + aac(6′)-aph(2″) + aadD/30, ant(6)-Ia_aph(3″)-III/20, ant(9)-Ia/14, aadD + ant(9)-Ia/9, ant(9)-Ia + aac(3)-XI-aac(6′)-aph(2″) + aadD/1, ant(6)-Ia + aadD + aac(6)′-aph(2″)/1, aac(6′)-aph(2″)/2, aph(3″)-III/2, aph(2″)-I1/1, aadD/3, ant(9)-Ia + aac(6′)-aph(2′)/3,	OXA/100%, FOX/96.9%, CFZ/76.7%
Aminoglycoside	aadD + ant(9)-Ia/9aph(3′)-III + ant(6)-Ia + aac(6′)-aph(2″)/1	GEN 38.3%
	*erm*(A)/75, *erm*(C)/16, *lnu*(A)/3, *erm*(B)/16, *erm*(T)/1, lsa(E) + *lnu*(B) + *erm*(C)/1, *lun*(A) + *erm*(X) + *erm*(A)/1, *erm*(C) + *lun*(A)/2, *lun*(A) + *erm*(A)/26,	
Macrolide	*msr*(A) + *mph*(C)/1	ERY/80.6%
	*tet*(M)/76, *tet*(K)/19, *tet*(L)/1,	
Tetracycline	*tet*(M) + *tet*(K)/2	TET/55.9%, MIN/41.5%
Trimethoprim	*dfrG*/9	T/SUL/3.19%,
Fusidic acid	*fusC*/1, *fusB*/1	/
Fosfomycin	*fosB*4/2	/
Phenicol	*cat*(pC233)/3, *fexA*/1	/
/	/	RIP/2.12%, LNE/1.59%, DOX/28.7%, MXF/69.7%, TEC/0.53%
QAC	*qacA*/38, *qacB*/38	/

The FD resistance genes (*fusC* and *fusB*), fosfomycin resistance genes (*foxB*), and phenicol resistance genes (*cat* and *fexA*) were rare (*n* = 1, 1, 2, 3, and 1, respectively). Thirty-eight (19.4%) MRSA isolates carried *qac*A/B genes. Three MRSA isolates (1.53%) only carry the *mecA* gene.

Minimum inhibitory concentration testing showed that 2.12% of MRSA isolates were resistant to RIP, 1.59% to LNE, 28.7% to DOX, 69.7% to MXF, and 0.53% to TEC. However, the related resistance genes could not be observed.

### Minimum spanning tree based on core genome multilocus sequence typing of ST764 methicillin-resistant *Staphylococcus aureus* isolates

**FIGURE 3:**
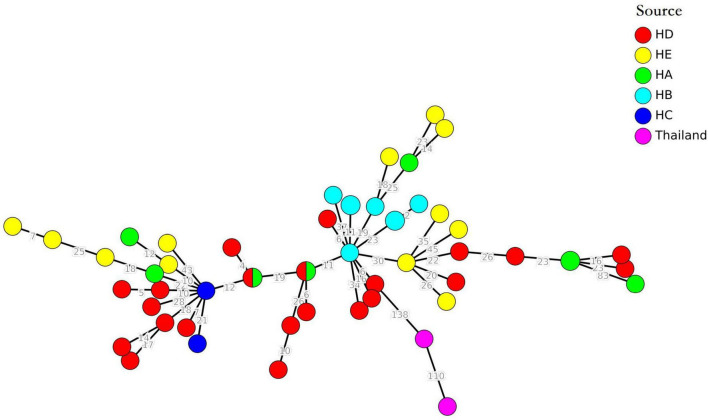
Minimum spanning tree of 51 ST764-MRSA-II isolates.

The cgMLST minimum spanning tree of 51 ST764 MRSA isolates was shown in Figure 3. Interestingly, only six clonal groups (≤8 allelic differences) were shown in Figure 3. Furthermore, two or three ST764 isolates were included in each clonal groups, and the clonal groups of ST764 isolates were identified in either the same hospitals or different hospitals. These results indicated the high diversity.

We expanded the database to include genomes of two ST764-MRSA-II isolates for Thailand from NCBI^5^ (Kondo et al., 2022). The minimum spanning tree showed that the allelic differences was more than 138, which suggested these two ST764 from Thailand were unrelated with the ST764 isolates from hospitals in Shanghai.

### Clone transmission of methicillin-resistant *Staphylococcus aureus* isolates in hospitals

**FIGURE 4:**
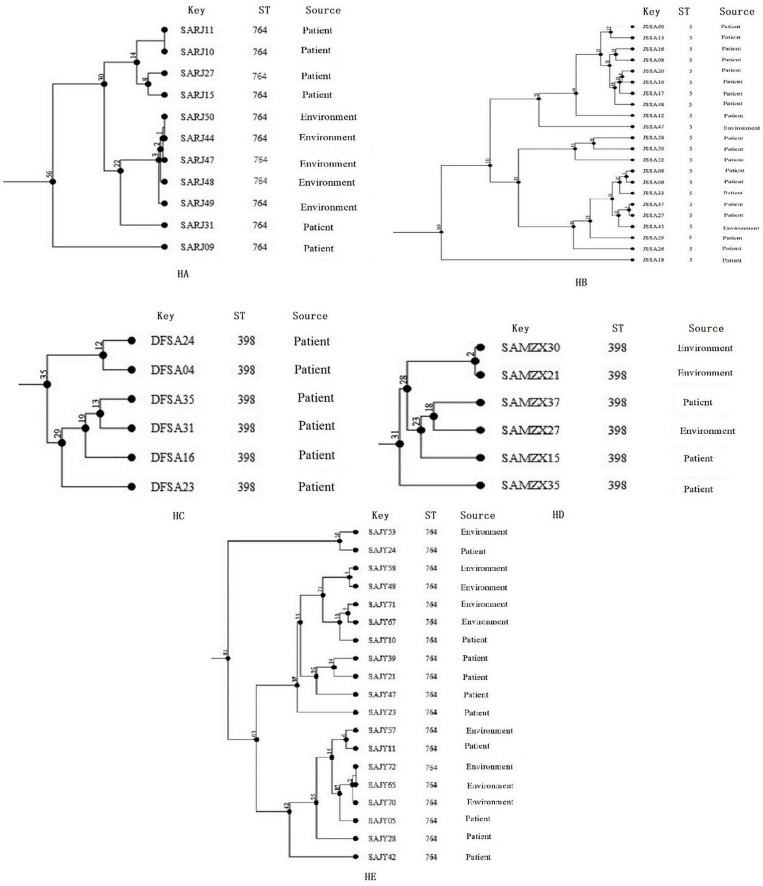
Clustering of partial Methicillin-resistant *Staphylococcus aureus* (MRSA) isolates from five hospitals, respectively, based on core genome multilocus sequence typing (cgMLST). The multiplication by one in the tree represents the number of different alleles among isolates. The corresponding data, including the name of these isolates (Key), ST, source (patient or environmental surface), and CL, are shown alongside the dendrogram to the right.

The diversity of cgMLST clusters of MRSA isolates is shown in Figure 4, and the 11 clonal groups (≤8 allelic differences) are shown in Figure 4. The clonal groups of MRSA isolates were mostly identified in the same hospitals and had several important characteristics. Firstly, the STs of clonal MRSA isolates were ST764, ST5, and ST398. Two clonal MRSA isolates in HA and HE were ST764, one clonal MRSA isolate in HD was ST398, and one clonal MRSA isolate in HB was ST5. No other STs of clonal MRSA isolates were found. Secondly, the clonal groups were identified among patients (four clonal groups), among environmental surface (four clonal groups), or among patients and environments (two clonal groups). For clonal groups of MRSA isolates identified in different environments, SAJY58 (doorknob) and SAJY48 (item in contact with patient) in HE showed four allelic differences in clone (CL) 8, SAJY71 (cupboard) and SAJY67 from (quilt) in HE showed five allelic differences in CL9. For clonal groups of MRSA isolates identified in both environmental surface and patients, SAJY57 (curtain) and SAJY11 (patient’s throat) in HE showed six allelic differences in CL10, and SARJ11 (blood) and SARJ10 (drainage) in HA showed no allelic differences in CL1. For clonal groups of MRSA isolates identified in different patients, JSSA37 and JSSA27 (both sputum) in HB showed three allelic differences in CL6, and JSSA09 (sputum) and JSSA06 (throat) showed five allelic differences in HB in CL5.

## Discussion

The five general hospitals chosen for this study included two upper first-class hospitals, two upper second-class hospitals, and one middle second-class hospital. These hospitals were chosen to obtain a better understanding of the prevalence of MRSA strains in Shanghai, China.

Our findings did not reveal any statistically significant difference between the proportions of CA-MRSA and HA-MRSA isolates. In this study, ST764, ST5, and ST398 were dominant in both HA-MRSA and CA-MRSA isolates in Shanghai, China, and ST398 has been reported as the predominant pathotype of CA-MRSA isolates in China (Ayesha et al., 2019; Jin et al., 2021). These results suggested that exchanges between the ecological niches of HA-MRSA and CA-MRSA clones might have occurred. The exchanges has been confirmed in other areas. The major HA-MRSA CC8-ST239 in South Korea, Hong Kong, and Taiwan and HA-MRSA CC5-ST5 in South Korea and Sri Lanka have traveled from hospitals into the community (Song et al., 2011). Therefore, the distribution between HA-MRSA and CA-MRSA isolates is becoming increasingly blurred. On the other hand, several studies have confirmed that a description of the genotypic and phenotypic characteristics for cases of exposure to a hospital environment for 48 h was insufficient (David et al., 2008; Otter and French, 2012).

According to our MLST results, a significant increase was observed in the incidence of ST764-t002-II MRSA infection, which was replacing ST5-t002-II MRSA as the predominant ST. Furthermore, ST5 MRSA was reported to decline during 2008–2018 in Shanghai, China (Jian et al., 2021), and ST764 was reported as a common genotype in *S. aureus* in blood samples during 2013–2018 in Shanghai (Jin et al., 2021). The ST764 clone is a common genotype in many settings in Japan and Thailand, including tertiary hospitals, outpatient departments, long-term care facilities, and communities, and even this genotype has been increasing (Aung et al., 2017, 2019; Kondo et al., 2022). Furthermore, ST764-MRSA-II isolates in Thailand might be brought by a healthy carrier for similar genetic characteristics (Kondo et al., 2022). However, these ST764-MRSA-II isolates in Thailand were not related with ST764-MRSA-II isolates in this study because there was more than 138 allelic difference among these them (Figure 3).

Recent studies have confirmed that ST764 was a new local variant of the globally disseminated ST5 lineage in Japan that acquired virulence traits, such as ACMEII and SaPInn54, which carry ACME *acrA* and the staphylococcal enterotoxin B gene (*seb*), enhancing the expression of cytolytic peptide genes (Takano et al., 2013). These similar ST764 isolates have been reported in Thailand. They shared the same genetic traits. All of them had *pvl*, *tst*, *sec*, *sel*, and *sep*, however, they did not have *seb* and *tet* (M) (Takano et al., 2013). The studies suggested that the ST764-MRSA-II clone might originate from the clone in Japan (Kondo et al., 2022). However, in our study, the ST764-MRSA-II clones were different from ST764-MRSA-II from Thailand and Japan. In our study, most ST764 (97.7%) carried *seb*, but not ACME *acrA* (Kondo et al., 2022). Furthermore, the minimum spanning tree confirmed that ST764-MRSA-II in Shanghai were unrelated with ST764-MRSA-II in Thailand and Japan, because there was more than 138 allelic differences among them. However, our research has a limitation. Transcription or proteomics data of these ST764 and ST5 MRSA isolates could provide more genetic proof of virulence than our WGS data. Nevertheless, the potential danger of spreading necessitated rigorous surveillance of emerging ST764 MRSA isolates.

In our study, ST398 MRSA comprised a major proportion of isolates from patients and environmental surface. ST398 was reported firstly as livestock-associated MRSA, then it was isolated from a human patient in 2003 in Europe (van Cleef et al., 2011). Subsequently, ST398 isolates have been reported as causing infections in humans in the absence of livestock exposure (Uhlemann et al., 2012; Brunel et al., 2014). Our study findings indicated that ST398 MRSA isolates are dominant in both HA-MRSA and CA-MRSA, which broadens our knowledge of ST398. Rigorous surveillance of MRSA strains in China is imperative to arrest its potential spread.

Our study results indicated higher resistance to seven antimicrobials (OXA, FOX, ERY, CFZ, CIP, LEV, and MXF). Resistance to GEN (38.3%), TET (55.9%), and MIN (41.5%) was common. It is expected that the proportion of these antimicrobial-resistant MRSA isolates will increase because they carry a relatively high level of SCCmec (type II and III), which typically harbors several antibiotic resistance genes (Sahreena and Zhanga, 2018). Resistance to T/SUL, RIP, LNE, DOX, and TEC was low. Antimicrobial resistance genes *mecA*, *blaZ*, ant(9)-Ia + aac(6′)-aph(2″), *erm*(A), and *tet*(M) were common in this study. The phenotypic resistance to OXA and the presence of *mecA* were consistent (100%). However, MRSA isolates that were resistant to RIP, LNE, DOX, MXF, and TEC did not carry resistance genes but showed phenotypic resistance to the content of the antimicrobials, as would be associated with the carrying of resistance genes. This cannot be explained by the involvement of more than one resistance mechanism in bacteria, genetic mutation, or changes in gene expression due to the external environment. Therefore, further research is necessary to judge whether the presence of resistance genes can be used as an indicator in identifying resistance.

We used cgMLST to compare MRSA isolates from patients with those obtained from the environmental surface within the five hospitals in our study. We found a strong link between these strains. We identified eleven clonal clusters, which were found to be associated with interhospital dissemination. Moreover, within each hospital, MRSA isolates were from different sources, such as patients and environmental surface in CL9 and CL10; environmental surface in CL3, CL7, and CL8; and patients in CL1, CL2, CL4, and CL5, and CL6. These results indicate that clonal transmission of MRSA isolates has occurred among environmental surface, among environments and patients, and among patients. The clonal relationship between equipment and patients infected with MRSA in hospitals has been confirmed (French et al., 1998; Griffifiths et al., 2002). Furthermore, most patients (69.8%) with serious disease were aged >60 years, making them more susceptible to infection by MRSA. Additionally, most MRSA isolates (45.1%) were from items that came into contact with patients (*P* < 0.05). Although it has not been confirmed whether clinical MRSA isolates isolated were in direct contact with these items, outbreaks caused by MRSA directly linked to an environmental source have been reported (Bures et al., 2000; Oie et al., 2002). Therefore, effective and regular cleaning and disinfection should be performed to prevent environmental contamination and nosocomial infections.

## Conclusion

The continuous surveillance of five hospitals in Shanghai, China, during 2017–2019 showed that the highest number of cases infected by MRSA isolates occurred in patients aged ≥60 years. A total of 19 STs of 162 MRSA isolates were identified from patients. A significant increase was observed in the incidence of ST764-t002-II MRSA infection, which is replacing ST5-t002-II MRSA as the predominant ST. Similarly, ST764-t002-II isolates from environmental surface were predominant. Most ST764 isolates carried *seb*, but not ACME-related genes. There was a high level of resistance to five antimicrobials (ERY, CFZ, CIP, LEV, and MXF). Resistance to GEN, TET, and MIN were also common. Phenotypic resistance to antimicrobials was associated with resistance genes to its content. cgMLST clusters suggested a strong link between these strains.

This study provides fundamental evidence for preventing and controlling clonal transmission of MRSA isolates in hospitals. Our findings suggest the application of WGS to monitor the changing epidemiology of MRSA isolates.

## Data availability statement

The datasets presented in this study can be found in online repositories. The names of the repository/repositories and accession number(s) can be found in the article/Supplementary material.

## Author contributions

HZ, LT, and ZF: methodology. WC, JB, and TC: software. HZ and LT: resources. YG: data curation. HZ: writing—original draft preparation. MC: funding acquisition. All authors have read and agreed to the published version of the manuscript.

## Footnotes

1
www.mgc.ac.cn

2
http://srs.genesclouds.com.cn/Home/AdminDefault

3
https://card.mcmaster.ca/

4
https://cge.cbs.dtu.dk/services/ResFinder/

5
https://www.ncbi.nlm.nih.gov/sra/?term=SRR
